# Awareness and factors associated with improved cookstove choice among end users in Kericho County, Rift Valley, Kenya

**DOI:** 10.1038/s41598-026-75061-7

**Published:** 2026-11-02

**Authors:** Samuel Adekunle, Obuks Ejohwomu, Nwabueze Emekwuru, Oluwapelumi Abiodun, Martie van Tongeren, Amanda Lea-Langton, Edwin Kimutai Too, Brexidis Mandila, Patrick Wamalwa, Zacharie Tamainot-Telto

**Affiliations:** 1 Faculty of Science and Engineering, The University of Manchester, Manchester, UK; 2 Civil Engineering Department, William VS Tubman University, Harper, Liberia; 3 CIDB Centre of Excellence, University of Johannesburg, Johannesburg, South Africa; 4 Department of Engineering, Harper Adams University, Newport, UK; 5 Faculty of Medicine, Biology and Health, University of Manchester, Manchester, UK; 6 University of Kabianga, PO Box 2030-20200, Kericho, Kenya; 7 Faculty of Engineering, Egerton University, Njoro, Kenya; 8 School of Engineering, University of Warwick, Coventry, UK

**Keywords:** Sustainability, Household cooking, ICS selection, Clean cooking, Traditional stoves, ICS adoption, Anthropology, Cultural and media studies, Cultural and media studies, Development studies, Environmental social sciences

## Abstract

The understanding of the user engagement, selection and adoption of Improved Cooking Stoves (ICS) in developing countries is important to deploy measures to sustain their use. As part of a project developing, deploying, and empowering communities in developing countries with ICS, this study screened the awareness and choice selection factors for end users selected for ICS adoption. This was achieved through a survey of 1200 households in Kericho County in the Rift Valley, Kenya. The results showed that, despite some awareness of ICS benefits, the traditional and semi-traditional stoves remain dominant (with about 70% in use against 30% of ICS) and are still adopted due to socio-cultural behaviour as well as low financial resources and settings. The study also revealed that 34.6% of end users use cookstoves for functions beyond their primary food preparation role, mainly for space heating, lighting and meat roasting. This is an important and hardly explored factor that should be considered in the design of ICS and the implementation of measures to improve ICS penetration in households in developing countries.

## Introduction

Cooking is a fundamental daily activity that sustains lives, yet in many regions, it relies on traditional biomass fuels and inefficient stoves, posing critical health and environmental risks. Globally, over 2.1 billion people depend on solid fuels such as firewood, charcoal, crop residues, and dung for cooking^1^. These traditional cooking methods often use open fires or rudimentary stoves, producing harmful emissions, degrading ecosystems, and imposing economic burdens.

The incomplete combustion of biomass fuels releases harmful pollutants such as carbon monoxide (CO), particulate matter (PM_2.5_), benzene, and formaldehyde, which have been linked to acute and chronic respiratory diseases, cardiovascular conditions, and adverse pregnancy outcomes^1–3^. The WHO estimates that exposure to household air pollution from solid fuel use contributes to approximately 2.9 million premature deaths annually, making it one of the leading environmental risk factors for mortality^1^. Women and children, who spend more time in the kitchen, bear the brunt of this burden, experiencing the highest levels of exposure.

Beyond health concerns, traditional cooking practices pose significant environmental and economic challenges. Collecting firewood and other biomass fuels contributes to deforestation and land degradation, particularly in regions with high fuelwood dependence, such as sub-Saharan Africa and South Asia^4^. This exacerbates climate change by increasing carbon dioxide (CO₂) emissions and black carbon deposition, accelerating global warming. Furthermore, reliance on inefficient stoves increases fuel consumption, placing additional strain on households, particularly low-income families who spend a disproportionate amount of their income on fuel. In urban areas, where charcoal is a predominant cooking fuel, high fuel prices create financial burdens for poor households^5^.

In response to these challenges, the development and promotion of Improved Cookstoves (ICS) have gained significant momentum over the past two decades. ICS are designed to enhance energy efficiency, reduce emissions, and minimize fuel consumption, addressing health and environmental concerns. Despite the potential benefits of these stoves, their adoption remains uneven and slow, particularly in rural and low-income communities.

The objectives of this study are to critically examine end users’ awareness and selection/adoption perceptions to produce actionable insights to improve intervention strategies. By focusing on end-user awareness and experience, this research aims to contribute to the ongoing discourse on clean cooking solutions and inform policies that better address community needs. It should be noted that this is a baseline study as part of a larger study to develop, deploy, and empower communities on improved cooking stoves in the global south. The research method is based on a survey carried out in Kericho County (Kenya), where firewood is predominantly used.

## Literature review

### ICS adoption

A critical review of ICS programs suggests that while millions of stoves have been distributed globally, sustained use is often low, with many households reverting to traditional cooking methods despite receiving new stoves^6^. This discrepancy between distribution and sustained use raises critical questions about the effectiveness of the dissemination strategies and the underlying factors that influence adoption. One of the primary issues is a lack of awareness and knowledge. Many users are not fully informed about the benefits of ICS or the risks associated with traditional cooking methods. In some communities, “clean cooking” is poorly understood or not prioritized, as immediate concerns such as food availability and economic survival precede long-term health and environmental issues^7^. Moreover, misinformation and misconceptions about ICS, such as doubts about performance, durability, and safety, might further hinder adoption.

Bailis et al.^4^ found that replacing traditional stoves with ICS could reduce wood consumption by up to 60% and cut greenhouse gas emissions by 40–80%. Similar findings were reported by Rosenthal et al.^5^, who observed that ICS projects in Kenya led to substantial reductions in carbon monoxide emissions, with improved rocket stoves and LPG stoves performing particularly well. However, the long-term environmental impact of ICS depends on sustained use and fuel availability. While ICS reduces deforestation pressure, many users revert to charcoal and firewood during fuel shortages, negating some ecological gains. Additionally, the production and disposal of certain ICS models, particularly those with metal components, raise concerns about lifecycle emissions and waste management^8^.

### Dynamics inherent in ICS adoption

Existing literature identifies socio-economic variables for instance income, education, household size, and age as primary determinants of ICS adoption. For instance, recent empirical work demonstrates that these variables significantly influence adoption decisions, with higher income and education levels positively associated with uptake^9^. However, a critical limitation of this body of work is its over-reliance on econometric explanations, often treating households as rational economic agents. While income constraints undoubtedly affect affordability, evidence suggests that socio-economic variables alone cannot explain adoption patterns. For example, even in contexts where stoves are subsidised or freely distributed, adoption and sustained use remain low^10^.

Furthermore, socio-economic analyses often neglect intra-household dynamics, particularly gendered decision-making. Although female-headed households have been associated with higher adoption rates, this relationship is mediated by broader socio-cultural norms and power relations, which are insufficiently theorized in existing studies.

Behavioral and cultural factors are underexplored determinants of cookstove adoption. Recent studies demonstrate that motivations, perceptions, and social norms significantly influence adoption decisions, often outweighing economic considerations^11^. For instance, cultural preferences related to taste, cooking methods, and social identity can inhibit adoption even when improved stoves are available and affordable^12^.

Moreover, social influence plays a pivotal role. Households are more likely to adopt ICS when neighbors or community members do so, highlighting the importance of peer effects and social learning. Despite this, many interventions continue to prioritize individual-level adoption strategies, neglecting community dynamics.

Lastly, structural conditions such as market access, supply chains, financing mechanisms, and policy frameworks play a decisive role in shaping adoption outcomes. High upfront costs, limited credit access, and weak distribution networks are consistently identified as barriers^9^. Additionally, the sustainability of adoption is closely tied to institutional trust and policy support, including subsidies and regulatory frameworks^12^.

## Research methodology

This study adopted a quantitative research design to analyze the awareness, adoption, and end-user experience regarding improved cookstoves (ICS) in Kenya. A descriptive cross-sectional survey was conducted to gather objective data at a single point, allowing for statistical analysis and identification of patterns based on the study objectives. The study was conducted in three selected sub-counties in Kericho County in Kenya: Belgut, Kipkelion East, and Soin Sigowet, covering 1200 households chosen randomly from the community empowerment groups. The data set was subjected to data screening and cleaning, dropping 49 observations because they are unfit for analysis. These locations were purposefully selected due to their diverse socio-economic, geographical, and cultural characteristics. Kericho County is located between longitude 35°02’ and 35°40’ East and between the equator and latitude 0°23’ South. Its altitude is about 2002 m above sea level with annual minimum and maximum temperatures of 10 °C and 25 °C respectively. The county’s main economic activity is agriculture, which is dominated by farming of tea, sugar, and coffee. Some peculiar meetings and terms to this area are highlighted below:

- Chama meetings: Gatherings of informal groups, often women, in Kenya and other parts of East Africa, who pool their resources for investment or other purposes.
- Chief barazas: Community meetings, often led by a chief or traditional leader, where local issues are discussed and resolved.

The survey examined the benefits and barriers associated with the uptake of fuel-efficient cookstoves in rural communities in Kenya, focusing on Belgut, Kipkelion East, and Soin Sigowet. A stratified random sampling approach was employed to ensure representation across gender, income levels, and urban-rural settings. The survey instrument focuses on the respondents’ background and the various sections on awareness and selection factors. Similar approach for survey design has been adopted in existing quantitative studies^13,14^. The questionnaire comprised seven sections covering identification; socio-economic and demographic characteristics; cooking characteristics and fuel information; technical performance and preferences relating to cooking stoves; cooking information; institutional characteristics; and financing and sustainability models. The instrument was pilot tested using five community leaders and five community members. Their feedback on the content of the questionnaire assisted in reviewing the instrument for validity and credibility. Also, it improved the correctness and removed any perceived error in the instrument.

The questionnaire contained closed-ended and open-ended questions eliciting the participants’ responses. This approach offered the respondent options to tick from the closed-ended questions and freedom to provide their responses in their own words without any stifling through the open-ended questions. Thus, providing robust feedback from respondents. When language was a barrier, the questionnaire content was explained and translated (between Swahili and English) to the respondents with adequate care so that meaning was not lost in translation. The survey conductors and their assistants were knowledgeable local residents who understood and spoke the relevant local languages, including Swahili, thereby reducing the potential influence of English-language barriers on respondents’ understanding of the questionnaire. They therefore ensured no meaning was lost in translation by critically comparing the translated against the original through back translation, by exchanging manuscripts among themselves.

The questionnaire was administered using the KoboCollect application, and each respondent participated only once. The quantitative data was analyzed using the excel software with descriptive statistics, including frequencies and percentages, summarizing household characteristics and stove usage patterns. The result of the analysis was displayed in Tables, 3D column chart and cylinder bar charts, clustered bar chart and funnel-style ranking chart.

The qualitative data was analyzed thematically through an inductive approach. The thematic coding and analysis were conducted manually by the first and second authors. Thereafter, they compared their coding decisions and resolved differences through discussion and code standardisation to ensure consistency in the application and interpretation of the coding framework. The resulting coding and interpretation were subsequently reviewed by the other authors as an additional quality-assurance step. This process enhance and ensured analytical rigor and ensure consistency in the interpretation of the data. It should be noted that informed consent was obtained from all participants, and data confidentiality was maintained in compliance with the Declaration of Helsinki^15^.

## Study findings

### Status of the household heads

A significant majority (78.0% (*n* = 898) of household heads are married, reflecting stable family units. Single household heads account for 11.8% (*n* = 136), while widowed or widower heads represent 8.7% (*n* = 100). Divorced individuals comprise the smallest proportion at 1.4% (*n* = 16). This pattern emphasizes the predominance of married household heads, which could impact family dynamics and resource allocation. This is presented in Table 1.

**Table 1:** Household heads status.

Marital status	%
Married	78.0
Divorced	1.4
Widow/widower	8.7
Single	11.8

### Education level of the household heads

**Fig. 1:**
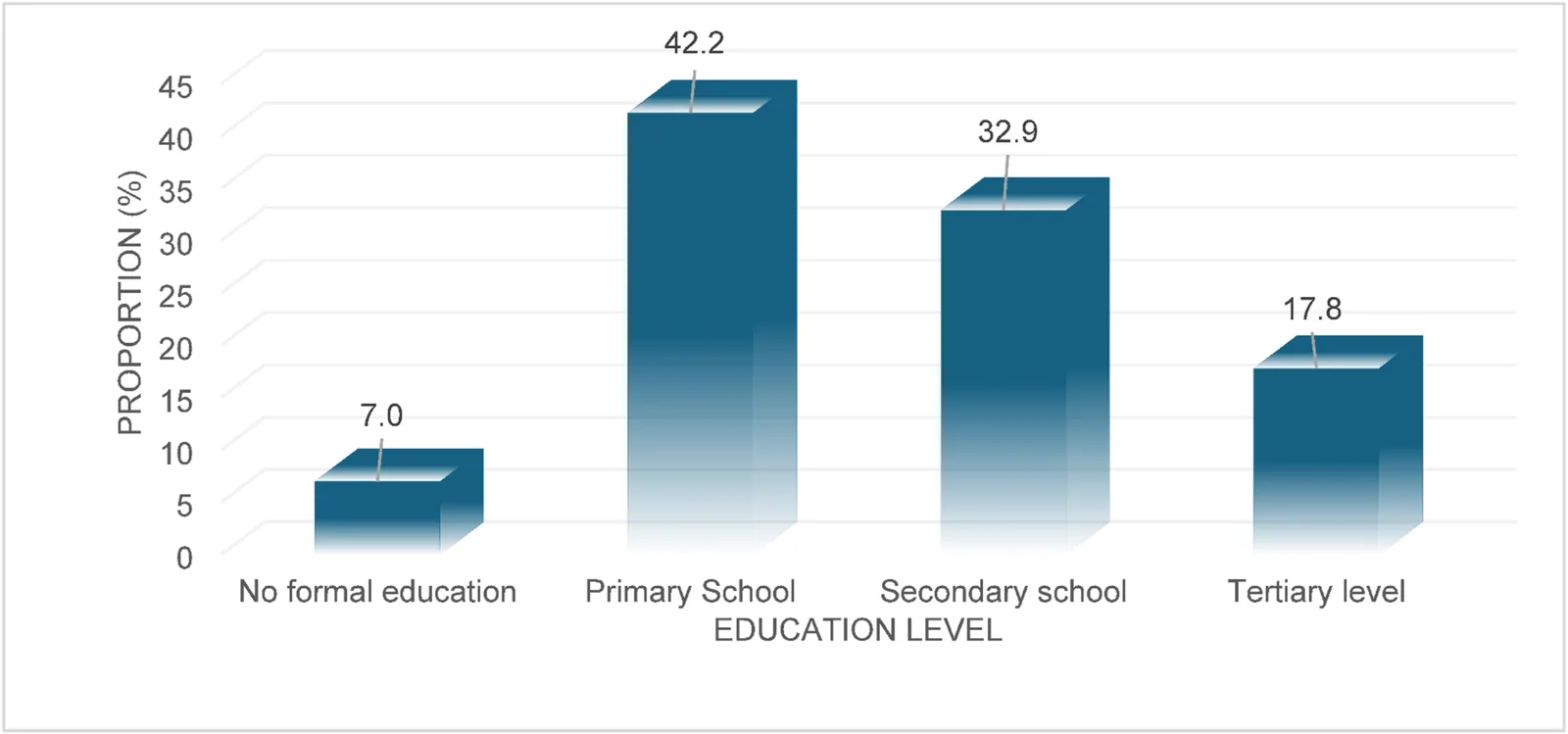
Education level of survey participants (Household heads only).

Education levels vary, as presented in Fig. 1, the most significant proportion (42.2%) have completed primary school. A considerable share (32.9%) has secondary education, while 17.8% attained tertiary-level education. A smaller group (7.0%) has no formal education. These findings suggest a relatively low higher education level among household heads, which could influence income-generating opportunities and household decision-making processes. In addition, this relatively low level of higher education could limit awareness and understanding of ICS’s benefits and the ability to access and interpret information about new technologies^7^.

### Household Size

The collected data indicates that the average household size is 5 members, with a standard deviation of 2. The smallest household comprises one member, while the largest has 13 members. This distribution reflects moderate household sizes, typical in many rural or semi-urban areas where extended families may reside together^16^. This is a critical factor that informs the design of the suitable cookstove to cater to the cooking needs of the entire household sufficiently.

### Health issues related to cooking

**Fig. 2:**
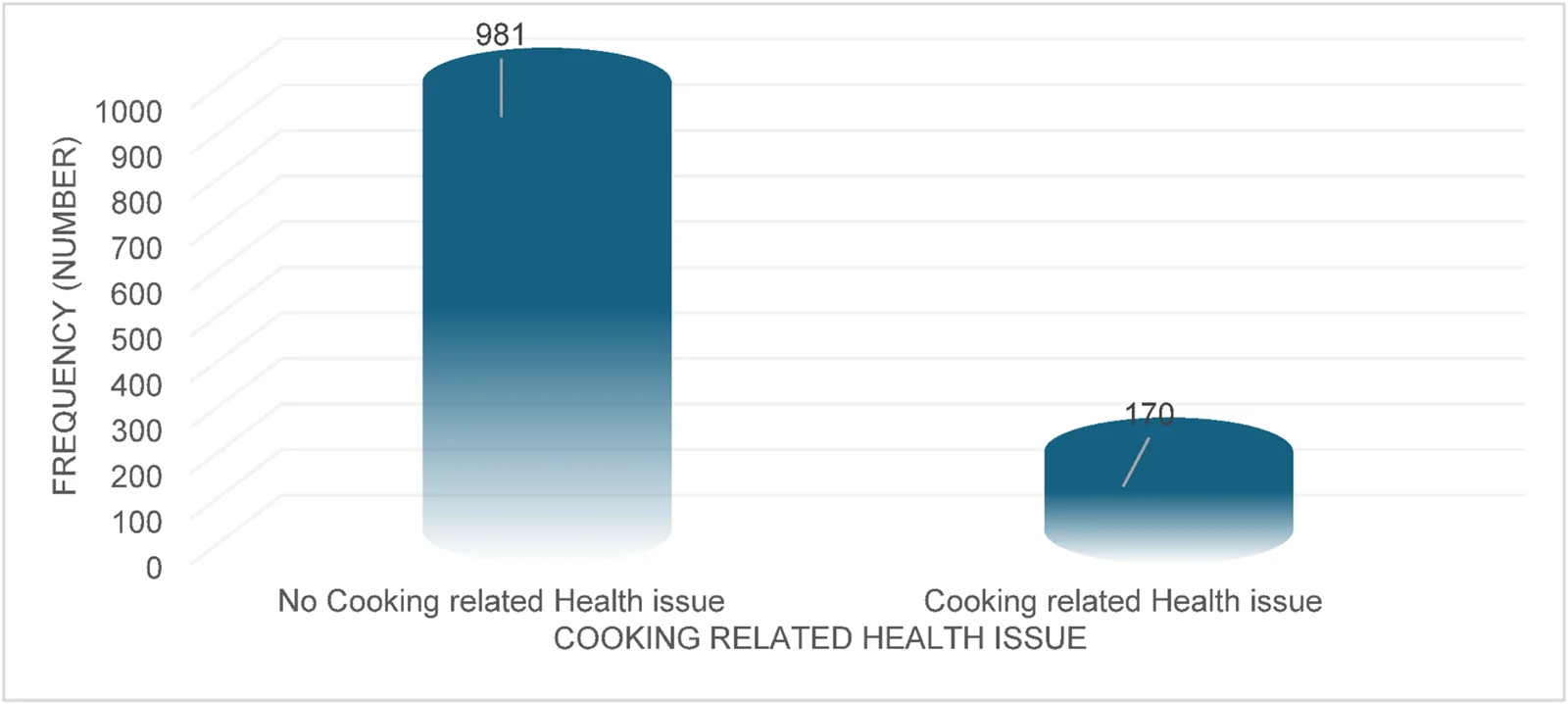
Health Issues related to cooking.

Cooking with cookstoves and other unsustainable options affects the end users negatively and health-wise. Figure 2 presents the responses of end users on the impact of the mode of cooking on their health. Most end users reported that their health was negatively impacted by cooking mode usage. Table 2 presents the responses on whether respondents’ health has been impacted or worsened.

**Table 2:** Health impacts related to cooking.

	Frequency	%
No	981	85.2
Yes	170	14.8

Regarding the health impacts of cooking methods, 85.2% (*n* = 981) of respondents reported no household members affected, while 14.8% (*n* = 170) reported some health impacts. **Table 2** suggests that the participants believed that many health challenges are directly linked to cookstove emissions or inefficiencies, highlighting the critical importance of interventions to improve cooking technologies and reduce smoke exposure.

170 households (14.77%) reported that at least one member is affected health-wise due to the cooking method. The health issues mentioned include (Table 3):

- Red eyes/Itching eyes: The most reported issue, affecting 71.63% of respondents experiencing health issues.
- Coughing (Pneumonia): Reported by 13.48%.
- Nose and throat problems: Reported by 12.77%.
- Asthma and Burns: Rarely reported, at 0.71% and 1.42%, respectively.

These health issues indicate exposure to smoke and inefficient cooking methods, emphasizing the need for cleaner cooking technologies and practices.

**Table 3:** Bodily reactions related to cooking.

Health issue	Frequency
Nose and throat problems	18
Coughing (Pneumonia)	19
Red eyes/Itching eyes	101
Asthma	1
Burns	2

These findings are consistent with previous studies that link indoor air pollution from biomass fuels to respiratory and ocular health problems (Smith et al., 2019). The high prevalence of health issues underscores the urgent need for cleaner cooking technologies to mitigate these adverse health effects^17^.

### Prevalent cookstoves used with firewood

**Fig. 3:**
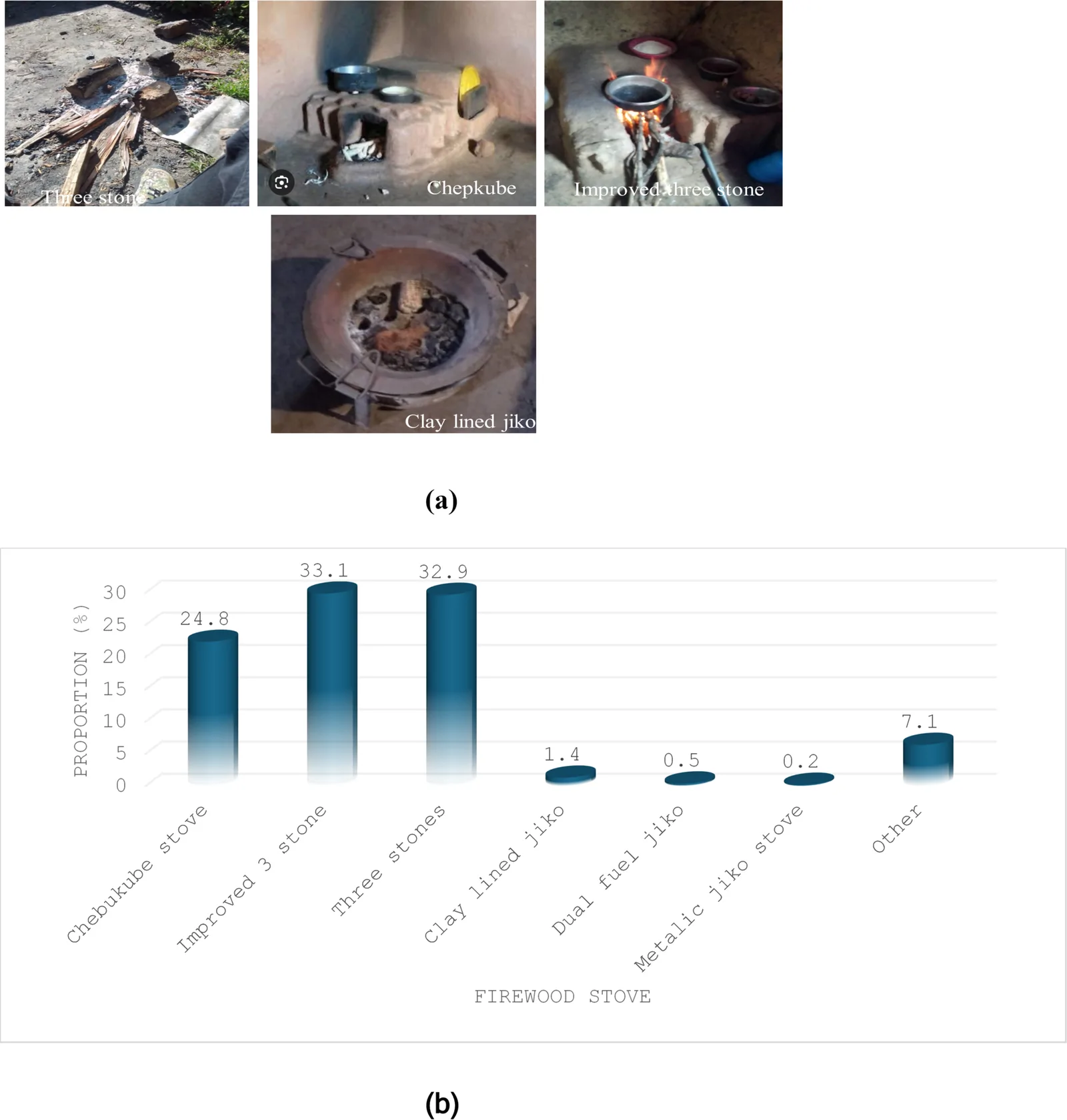
(**a**) Pictures of prevalent stoves, (**b**) types of stoves used with firewood.

Figure 3a shows how the stoves look in real life. All households that use firewood but adopts different stove types, as illustrated in Fig. 3b, the Improved 3-Stone Stove is the most used, with 33.1% of respondents reporting its use. This is closely followed by traditional Three-Stone Stoves (32.9%), highlighting the persistence of traditional cooking methods. The Chebukube Stove (24.8%) also has notable adoption. Other stoves, such as the Clay-Lined Jiko (1.4%), Dual-Fuel Jiko (0.5%), and Metallic Jiko (0.2%), are much less commonly used. 7.1% of the households use other types of traditional cookstoves. These findings indicate that while there is some shift toward improved stoves, traditional and semi-traditional stoves remain dominant.

### Sources of information on cookstoves and bioenergy

To adequately understand the age-long preference for traditional cooking methods, the study sought the qualitative response of the end users on the source of information regarding cookstoves and bioenergy. This explores the source of education of the end users regarding cookstoves. Table 4 presents the emerging themes from their responses. It is interesting to note that most respondents get their information from family/friends and neighbors. Thus, it is easy to believe the information they gather as it is coming from trusted sources. However, the authenticity and relevance of this information are not explored further. Other sources include radio and chama meetings. Chama meetings refer to self-organized economic support networks, commonly identified as community lending circles, where members pool funds to save, access loans, and pursue small-scale investments together. It should, however, be noted that the latter two do not gather the same number of responses as the first.

**Table 4:** Sources of information.

Information source	Proportion (%)
Chama meetings	17.19
Chief barazas	2.87
County fora	0.15
Family/friends/neighbours	40.87
NGOs	3.77
Social media	5.28
Radio	18.70
TV	3.62
Other	7.54

### Critical selection factors for ICS

**Fig. 4:**
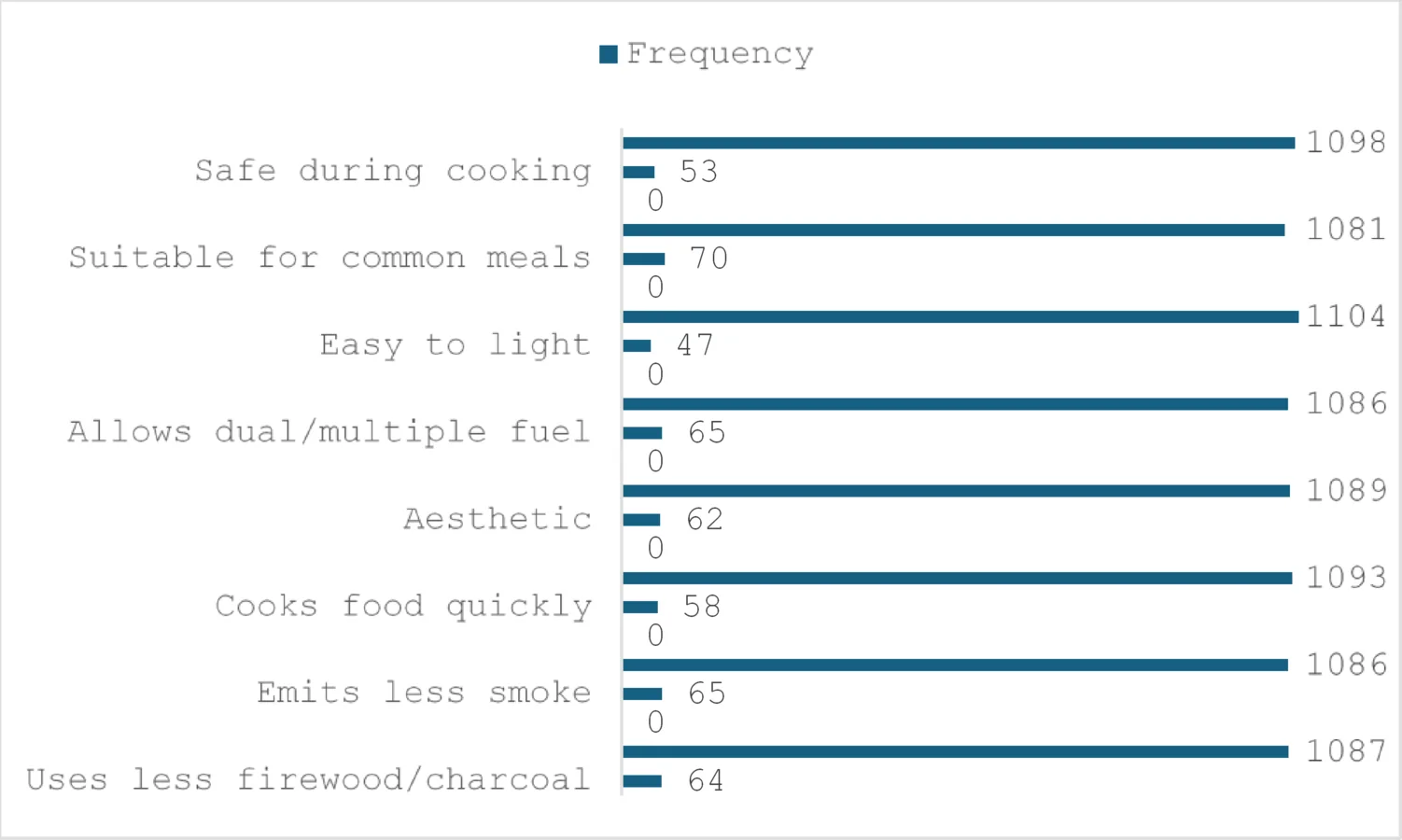
Individual factors rating.

To achieve this objective, respondents were asked first to state if each variable would be considered a factor in adopting an ICS. Afterwards, they were asked to rate the variables in order of significance. Figure 4 shows that all factors are significant as respondents answered affirmatively; however, the level of affirmative responses is not the same. From the figure the number of respondents who answered affirmatively to these factors are represented by the long bars while the short bars represented the number of negative responses.

**Fig. 5:**
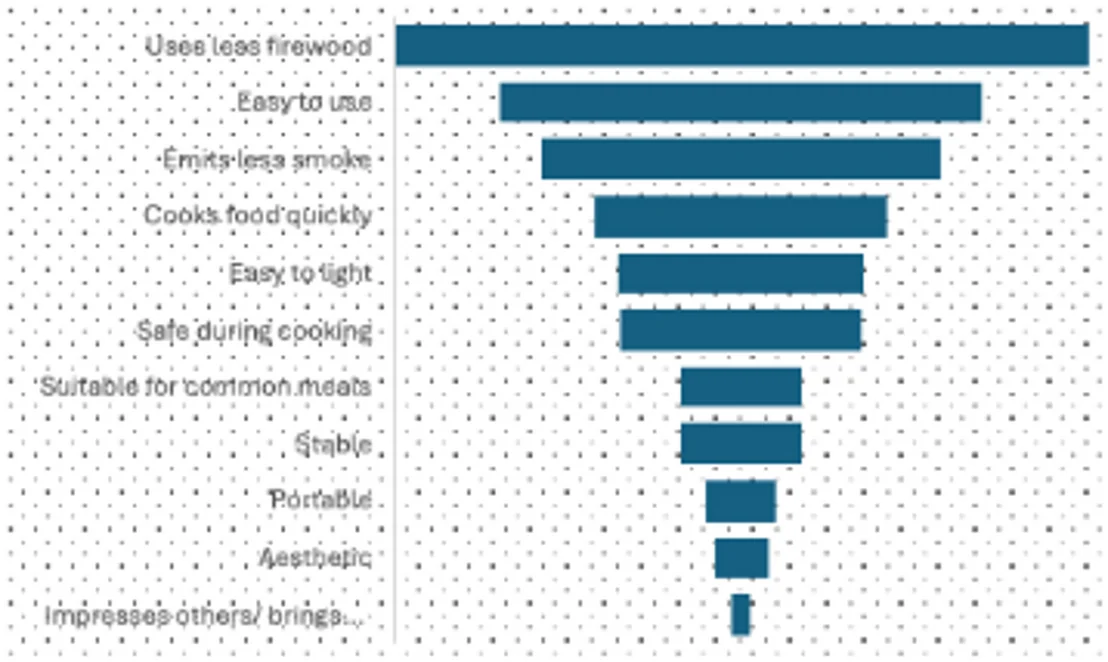
Critical ranking of selection factors.

The end users ranked these factors cumulatively, as presented in Fig. 5. It is observed that “uses less firewood” was ranked first by the respondents. This is followed by “easy to use”, “emits less smoke”, and “cooks food quickly”. A glance at the table suggests efficiency, sustainability and ease of use. Meanwhile, respondents are not particular about aesthetics and social outlook.

### Other uses of cookstove

The multifunctionality of cookstoves beyond their primary role in food preparation is a critical yet often underexplored dimension in household energy use. As shown in Table 5, respondents identified a range of secondary uses for cookstoves, including space heating (20.3%), lighting, and meat roasting. More importantly, 34.6% of households use the cookstove for other purposes than the primary function of cooking.

**Table 5:** Other uses of cookstoves.

Other household uses of the cookstove	Frequency	%
Only for cooking	752	65.4
Space heating	234	20.3
Other	64	5.6
Space heating; lighting	33	2.9
Roasting meat; space heating	23	2
Roasting meat	19	1.6
Lighting; space heating	7	0.6
Roasting meat; space heating; lighting	6	0.5
Space heating; roasting meat	5	0.4
Lighting	4	0.3
Roasting meat; lighting; space heating	3	0.3
Space heating, roasting meat, lighting	1	0.1

## Discussions

The result of the study is discussed in this section. The findings of this study highlight a significant gap between the awareness of clean cooking benefits and the actual adoption of Improved Cookstoves (ICS) in Kericho County. While global interventions often focus on technical efficiency, the dominance of traditional and semi-traditional stoves (70%) (Fig. 3b) in this region underscores that socio-cultural behavior and economic constraints remain the primary drivers of stove selection. This suggests that end-users prioritize familiar, reliable methods over new technologies, even when they acknowledge the associated health risks like respiratory issues and ocular irritation.

Despite the availability of various ICS models, traditional and semi-traditional stoves remain dominant and widely adopted. The Improved 3-Stone Stove and traditional Three-Stone Stoves are the most used, indicating a slow transition to more efficient cooking technologies. This persistence of traditional methods can be attributed to several factors, including cultural preferences, economic constraints, and the perceived reliability of traditional stoves as already pointed out by Bensch and Peters^18^. End users seem to trust the traditional ways of doing things more than new ways. The study’s findings suggest that while there is some awareness of ICS, significant barriers to adoption remain; this might be due to economic accessibility and cultural adaptability. Hence, more work is required on intentional and strategic approaches to macro-level awareness and adoption.

The primary sources of information about cookstoves and bioenergy were family, friends, and neighbors, followed by radio and community meetings. This reliance on informal networks highlights the importance of social influence and peer learning in the dissemination of information about ICS^19^. However, the authenticity and accuracy of information from these sources may vary, potentially affecting the perceptions and adoption of ICS. The current study underscores the need for targeted awareness campaigns and educational programs to provide accurate and comprehensive information about ICS’s benefits and proper use^7^.

Furthermore, the study identified key factors influencing the adoption of ICS, with “uses less firewood” being the most critical, followed by “easy to use,” “emits less smoke,” and “cooks food quickly.” These priorities reflect the practical and economic considerations of end-users, who seek to reduce fuel consumption and cooking time while minimizing smoke exposure. The emphasis on efficiency and ease of use aligns with previous research, indicating that these attributes are crucial for the sustained adoption of ICS^19^. However, aesthetics and social prestige were deemed less important, suggesting that functional benefits and sustainability outweigh social considerations in the decision-making process.

The data also reveal that space heating is the most prevalent secondary use, reported by 20.3% of respondents. This high proportion suggests that households rely on cookstoves for cooking and as a vital source of ambient heat, particularly in colder regions or seasons. This finding aligns with studies in similar contexts where cookstoves address multiple domestic energy needs due to the absence of alternative heating technologies (e.g.^8,20^).

Other secondary uses include lighting and roasting meat, although these are much less common. Only 0.3% to 2.9% of respondents reported combinations of these uses, with roasting meat alone accounting for 1.6%. The relatively low use of cookstoves for lighting may be attributed to increasing access to alternative lighting sources, such as solar lanterns and grid electricity in some areas, or the inefficiency of open flames for illumination.

The small proportions of households using cookstoves for multiple combined purposes, such as space heating, lighting, and roasting meat (ranging from 0.1% to 2.9%), indicate a subset of users who view the stove as a multifunctional household asset. These uses may reflect economic rationality, where households aim to maximize the utility of limited energy resources^21^. Thus, many current ICS designs, which focus narrowly on thermal efficiency for food preparation, may be perceived as technically superior but functionally inferior in a holistic household context. If a new stove cannot provide the ambient heat required in the cooler climate of Kericho, users are economically and practically rational to revert to traditional three-stone fires. This bridges the study’s findings with the broader field by arguing for user-centred design that accounts for the “stove stacking” phenomenon^22^ and the diverse energy needs of rural households.

### Implications of the study

The findings of this study have significant implications for policy and practice. To enhance the adoption of ICS, interventions must address the identified barriers, including economic constraints, cultural preferences, and information gaps. Financial support mechanisms, such as microloans and subsidies, could make ICS more accessible to low-income households^8^. Integrating behavioral change strategies with technological innovations is essential to ensure sustained use and maximize health and environmental benefits^17^. Policymakers should also consider the role of social networks and community-based approaches in promoting ICS adoption, leveraging trusted sources of information to enhance awareness and acceptance^19^.

The data underscore the importance of understanding user behaviour and multifunctional expectations in cookstove design and dissemination. Cookstove interventions that fail to account for secondary uses risk low adoption or premature abandonment, as users may revert to traditional stoves that better meet broader household needs^17^.

Design innovations should, therefore, consider integrating or enhancing features that support space heating or roasting, especially in regions with cooler climates or where meat roasting is culturally significant. For instance, stoves with heat-retaining materials or those that enable horizontal flame dispersion could improve efficiency for heating purposes without compromising cooking performance.

Furthermore, recognizing these additional functions has implications for impact assessment. Evaluating cookstove interventions solely on cooking-related outcomes may underestimate their role in household energy systems, thereby overlooking potential benefits or unintended consequences associated with reduced access to heat or lighting if traditional stoves are removed. This is a critical factor to be considered in developing ICS as it not only improves ICS adoption but by extension impacts energy management and climate change.

### Limitations and future directions

While comprehensive, this study is limited by its cross-sectional design, which provides only a snapshot of adoption rather than tracking long-term sustained use. Additionally, the reliance on self-reported data may introduce biases regarding stove usage patterns. Future research should employ longitudinal methods and objective fuel-use monitoring to validate these findings and explore how adoption dynamics evolve as households transition through different socio-economic stages. Ultimately, achieving a sustainable energy transition in the Global South requires an integration of technological innovation with deep behavioural and cultural insights.

## Conclusion

This study provides a comprehensive analysis of the awareness and choice selection factors of end-users for innovative cookstoves (ICS) in a developing country context, specifically focusing on Kenya. The study revealed that demographic factors, such as marital status and education levels, play a crucial role in adopting ICS. The predominance of married household heads and the relatively low levels of higher education among respondents suggest that stable family units and limited educational attainment may impact decision-making processes and the prioritization of household needs.

The study identified key factors influencing the adoption of ICS, with efficiency, ease of use, and reduced smoke emissions being the most critical. These priorities reflect the practical and economic considerations of end-users, who seek to reduce fuel consumption and cooking time while minimizing smoke exposure. The reliance on informal networks, such as family, friends, and neighbors, for information about cookstoves and bioenergy highlights the importance of social influence and peer learning. The study underscores the need for targeted awareness campaigns and educational programs to provide accurate and comprehensive information about ICS.

The findings demonstrate that many households utilize cookstoves for more than cooking, particularly for space heating. This multifunctionality must be factored into cookstove design, dissemination, and evaluation strategies. Addressing these broader household energy needs may enhance the relevance and uptake of improved cookstoves, ultimately contributing to more sustainable and acceptable energy transitions in low-resource settings.

By integrating technological innovations with behavioral change strategies and leveraging social networks, policymakers can promote cleaner cooking technologies and improve health and environmental outcomes. Future research should continue to explore these factors in diverse contexts to develop more effective interventions and policies that address the needs of communities in developing countries. Despite its comprehensive approach, the study has some limitations. Firstly, the cross-sectional design provides a snapshot of ICS adoption and usage patterns but does not capture changes over time. Longitudinal studies would be beneficial to understand the dynamics of adoption and sustained use. Secondly, the reliance on self-reported data may introduce bias, as respondents might overstate or understate their usage and experiences with ICS. Future research should consider diverse settings and larger sample sizes to validate and extend the results.

## Data Availability

In accordance with the University’s data protection policy, any data containing personal identifiers must be encrypted when not stored on secure University systems or kept as hard copy accessible only to the research team. To protect participant privacy and comply with the General Data Protection Regulation (GDPR), the raw data for this study are not publicly available. De-identified data may be provided on reasonable request to the corresponding author, subject to a data-sharing agreement and any required ethical approvals.
